# Hepatocyte growth factor secreted by bone marrow stem cell reduce ER stress and improves repair in alveolar epithelial II cells

**DOI:** 10.1038/srep41901

**Published:** 2017-02-03

**Authors:** Izabela Nita, Katrin Hostettler, Luca Tamo, Michaela Medová, Giuseppe Bombaci, Jun Zhong, Ramanjaneyulu Allam, Yitzhak Zimmer, Michael Roth, Thomas Geiser, Amiq Gazdhar

**Affiliations:** 1 Department of Pulmonary Medicine, University Hospital Bern, Switzerland; 2 Department of Clinical Research University of Bern, Switzerland; 3 Pulmonary Cell Research, Department of Biomedicine, University and University Hospital Basel, Basel, Switzerland; 4 Graduate school of Biomedical science, University of Bern, Bern, Switzerland; 5 Department of Radiation Oncology, Inselspital, Bern University Hospital, and University of Bern, Bern, Switzerland; 6 Department of Hematology, University Hospital Bern, Bern, Switzerland

## Abstract

Idiopathic Pulmonary Fibrosis (IPF) is a progressive, irreversible lung disease with complex pathophysiology. Evidence of endoplasmic reticulum (ER) stress has been reported in alveolar epithelial cells (AEC) in IPF patients. Secreted mediators from bone marrow stem cells (BMSC-cm) have regenerative properties. In this study we investigate the beneficial effects of BMSC-cm on ER stress response in primary AEC and ER stressed A549 cells. We hypothesize that BMSC-cm reduces ER stress. Primary AEC isolated from IPF patients were treated with BMSC-cm. To induce ER stress A549 cells were incubated with Tunicamycin or Thapsigargin and treated with BMSC-cm, or control media. Primary IPF-AEC had high Grp78 and CHOP gene expression, which was lowered after BMSC-cm treatment. Similar results were observed in ER stressed A549 cells. Alveolar epithelial repair increased in presence of BMSC-cm in ER stressed A549 cells. Hepatocyte growth factor (HGF) was detected in biologically relevant levels in BMSC-cm. Neutralization of HGF in BMSC-cm attenuated the beneficial effects of BMSC-cm including synthesis of surfactant protein C (SP-C) in primary AEC, indicating a crucial role of HGF in ER homeostasis and alveolar epithelial repair. Our data suggest that BMSC-cm may be a potential therapeutic option for treating pulmonary fibrosis.

Repeated micro-injuries to the alveolar epithelium contribute to pathogenesis of idiopathic pulmonary fibrosis (IPF), resulting in cellular dysfunction thus aggravating disease progression^1^. In response to injuries, the alveolar epithelium secretes pro-fibrotic and pro-inflammatory mediators which in turn activates fibroblasts and myofibroblasts, leading to increased synthesis and deposition of extracellular matrix (ECM)^2^. However, the underlying mechanisms that impair alveolar epithelial repair in response to injury are not fully understood. Recently, the role of intracellular organelles in the pathogenesis and progress of lung fibrosis had been elucidated^3^,^4^. Endoplasmic reticulum (ER) may be involved in several fibrotic diseases including IPF^5^. The ER is an intracellular organelle controlling Ca^2+^ homeostasis, synthesis, folding and maturation of most secreted and transmembrane proteins. Processes that disturb ER homeostasis lead to ER stress. ER stress is defined as an accumulation of misfolded or unfolded proteins in the ER lumen, and is induced by three signaling receptors: ATF6 (activating transcription factor-6), PERK (PRK-like ER kinase) and IREIα (inositol-requiring enzyme1α). In physiological conditions, these receptors are bound in an inactive form to the ER chaperone GRP78. Under ER stress, GRP78 is released from these compounds, and activates them. When activated, these receptors enhance protein accumulation and increase GRP78 and CCAATT/enhancer-binding protein-homologous protein (CHOP) expression, indicating ER stress^6^,^7^.

Recent evidence suggests that ER stress plays a novel direct role in the pathogenesis of IPF^8^,^9^. Initially, ER stress was reported in familial IPF due to mutation of surfactant protein C (*SFTPC*)^10^. However, recently, ER stress was also described in sporadic IPF independent of SFTPC mutations^11^. Furthermore, studies in animal models revealed the essential role of ER stress in acute lung injury^12^ and lung remodeling^13^. ER stress also plays an important role in epithelial-to-mesenchymal transition (EMT)^14^ and in alveolar epithelial cells (AEC) cell death^15^. Together, these data indicate that ER stress is a key regulator involved in the pathogenesis of acute and chronic lung injuries. Since ER stress mediates intracellular signaling pathways, it may be an essential therapeutic target to reduce or reverse tissue remodeling, stimulate epithelial repair, and initiate tissue regeneration in the fibrotic lung. In a recent study, we showed that bone marrow-derived stromal cells (BMSC) overexpressing hepatocyte growth factor (HGF) attenuated bleomycin-induced fibrosis in rat lungs^16^. BMSC treatment has been applied in animal studies and some clinical trials^17^, including for treatment of pulmonary fibrosis^18^. Interestingly, during *in vitro* culture, BMSC secrete a range of cytokines and growth factors, including KGF, Ang-1 and HGF, which have a biological effect^19^,^20^. Various disease models have explored secreted mediators from BMSC as potential therapeutic option^21^,^22^,^23^. But the effect of these secreted mediators on alveolar epithelium of fibrotic lungs and on endoplasmic reticulum balance has not been studied.

In the current study, we investigated the role of ER stress on the capacity of alveolar epithelium to self-repair using ER stressed A549 cells. Moreover, we studied the effect of BMSC secretome on ER stress response in primary AEC obtained from IPF patients and on ER-stressed A549. We also found BSMC-cm mediated improvement in wound healing in ER-stressed A549 *in vitro*.

## Material and Methods

### Ethical approval for patient material

Written informed consent was obtained by the University Hospital, Bern for paraffin-embedded lung tissue. Approval from the Human Ethics Committee of the University of Bern, Switzerland was also obtained for isolating human BMSC. The Human Ethics Committee of Basel (EKBB #05/06, Pneumology University of Basel, Switzerland) approved the process of isolating primary human AEC from tissue obtained from surgical lung biopsies. Written informed consent was obtained from all patients involved in the study. All procedures were performed in accordance with the relevant guidelines and regulations.

### Paraffin embedded lung tissue

We studied lung tissue sections from patients diagnosed with Usual interstitial pneumonia (UIP) and Idiopathic pulmonary fibrosis (IPF), according to ATS/ERS clinical guidelines^24^,^25^, and obtained formalin-fixed and paraffin-embedded lung wedge resections of patients with UIP/IPF from the Institute of Pathology, University of Bern, Switzerland.

#### Culture and characterization of primary human AEC from IPF patients

Primary human AEC were isolated from surgically removed lung biopsies by cell sprouting. Peripheral lung tissue was cut into small pieces of approximately 1 mm^3^, and placed into pre-wetted 25 cm^2^ cell culture flasks (Falcon, Corning Incorporated NY, USA) by 1 ml Epithelial growth medium (CELLnTEC, Bern, Switzerland) no additional coating was applied. The tissue was left in the cell culture incubator in standard conditions of (37 °C, 21% O2, 5% CO2) the epithelial growth media was replaced every fourth day. After approximately four to five days cell sprouting was observed from the edge of the lung tissue, the cells were allowed to grow till a confluent monolayer is achieved. When the cells reached confluence the experiments were performed, the cells were not passaged. Characterization of alveolar epithelial cells was performed using immunofluorescence staining for the epithelial marker E-cadherin and Surfactant Protein A (SP-A), and by immuno-fluorescence staining and relative mRNA expression of Surfactant Protein C. All experiments using the primary cells were performed in triplicate (n = 3).

### Isolation and characterization of human BMSC and generation of BMSC conditioned media

Human BMSC were obtained from the patients undergoing orthopedic surgery at the University Hospital Bern, Switzerland. The cells were grown in DMEM, supplemented with 10% FBS, fetal bovine serum (FBS) and 1% penicillin/streptomycin (P/S). To characterize the BMSC, cell surface immunophenotype was analyzed by staining the BMSC with phycoerythrin- or fluorescein isothiocyanate (FITC)-labeled monoclonal antibodies against CD29 (#561795), CD90 (#555595), CD45 (#560976), CD31 (#566177), CD106 (#555647) (BD biosciences, Allschwil, Switzerland), CD44 (#F10-44-2) (Serotec, Oxford UK). The cells were resuspended in PBS to obtain a single cell suspension and incubated for 45 minutes at 4 °C with the staining antibody. 20 μl FcR blocking reagent per 107 cells were added (Miltenyi Biotec, Cologne, Germany). Cells were washed three times in staining buffer (1% BSA and 0.1% NaN3 in PBS) to remove unbound antibody. The cells were then resuspended in staining buffer and analyzed with the FACS scan (BD Bioscience, Allschwil, Switzerland).

All experiments were performed at cell passage 2–3. When BMSC reached 80–85% confluence, they were washed thrice with 1x PBS, and were grown for 24 hours in DMEM with 1% P/S in absence of FBS. The cell culture medium was then collected, centrifuged (1.2 g) for 10 minutes, and used for experimentation as BSMC-cm.

### Reagents and Antibodies

Tunicamycin (TN), thapsigargin (TG), mouse anti-vimentin Cy 3 conjugated (#C5992), and mouse anti-pan cytokeratin FITC conjugated antibody (#C9080) were obtained from Sigma-Aldrich (St. Louis, MO). Anti-HGF affinity purified polyclonal antibody (#AF-294-SP) and recombinant HGF was purchased from R&D Systems (Abington U.K), and recombinant TGF-β1 was from MyBiosource Inc, (San Diego, USA).

### Cell lines

Human A549 alveolar epithelial-like cells (AECII) were obtained from the American Type Culture Collection (ATCC, Rockville, MD, USA), and were grown in DMEM supplemented with 10% fetal bovine serum (FBS) and 1% penicillin/streptomycin (P/S).

### ELISA

The human HGF Quantikine ELISA kit (R&D Systems, Abingdon, UK), was used to quantify HGF level in BMSC-cm. Following the instructions provided by the manufacturer.

### Inducing ER stress

A549 cells were grown to confluence and treated with either TN 2 μg/ml for 24 hours, or TG 0.1 μM for 7 days to produce ER-stressed AEC. These were used in various assays, as described below. For *in vitro* lung epithelial wound healing experiments, the cells were grown in 24 well plates; for RNA extraction, the cells were grown in 6 well plates. All experiments were performed in triplicates (n = 3).

### Neutralizing HGF in BMSC-cm

BMSC-cm was treated with 0.8 ng/ml of anti-HGF neutralizing antibody (R&D Systems, Abingdon, UK) (#AF-294-SP) 30 minutes at 37 degree Celsius and used for experimentsaccordingly to the manufacturer’s instructions. Goat IgG isotype antibody R&D Systems (Abingdon, UK) was used for isotype control experiments.

### Recombinant HGF treatment

ER stressed A549 cells were treated with 1.6 ng/ml recombinant HGF from R&D Systems (Abingdon, UK).

### TGFβ1 treatment of A549 cells

Monolayer of A549 cells were treated with 8 ng/ml of recombinant TGF-β1 (MyBioSource Inc, San Diego USA), and lysed at 6 hrs, 24 hrs and 48 hrs for RNA extraction. Nucleospin RNA extraction kit (Macherey-Nagel, Düren, Germany) was used to lyse for RNA extraction. Random hexamers primers were used for the reverse transcriptase polymerase chain reaction (RT-PCR) of 1 μg total RNA in a reaction volume of 20 μl; using Omniscript RT kit (Qiagen, Maryland USA). Real-time qRT-PCR was performed using the Quantitect Probe PCR kit with SYBER green reagent (Applied Bioscience, Foster City, CA USA).

### Immunohistochemistry

Formalin-fixed human lung tissue sections were de-paraffinized in a xylene series and rehydrated through a decreasing ethanol series for immunohistochemistry. The slides were pre-treated by microwave in citrate buffer (100 mM, pH 7.0) for 10 minutes, washed 3x with 1x PBS and 0.1% tween (TBS). Slides were incubated overnight at 4 °C in an anti-Grp78 antibody dilution (1:100) from Santa Cruz Biotechnology (Santa Cruz Biotechnology, Santa Cruz, CA, USA). EnVisionTM (DAKO, Bollschweil, Germany) was used for staining and detection, according to the manufacturer’s instructions. A Leica DMI 4000 D microscope was used to acquire images. For double immunofluorescence, tissue sections were incubated with primary antibodies specific to GRP78 (1:100) or SP-C (1:100) (surfactant protein C) (both: Santa Cruz Biotechnology, Santa Cruz, CA, USA). The secondary antibodies used were anti-goat Cy3 labelled to detect GRP78, and anti-rabbit FITC labelled to detect SP-C (Abcam, USA); both secondary antibodies were diluted 1:1000.

### *In vitro* alveolar epithelial wound repair assay

A549 cells were grown to confluence, and then treated with TN for 18 hours or TG for 7 days as described above to induce ER stress. A wound was mechanically created by scratching the cell layer with a pipette tip. After the cell medium was washed, it was exchanged for BMSC-cm or serum free media (negative control), or media with 10% serum that served as positive control. Images of the wound surface were captured at time 0 and after 24 hours with a Leica DMI 4000B. Image J software (NIH, USA) was used to analyze the wound surface area. Wound repair was expressed as the percentage of wound surface area that had closed after 24 hours.

### Immunofluorescence

Primary AEC isolated from lung biopsy were fixed in 3% paraformaldehyde (PFA) for 10 minutes at room temperature, washed three times in 0.1% glycin/PBS, and incubated with either rabbit polyclonal antibody to E-cadherin (1:200) (#ab53226) (Abcam, USA), or rabbit polyclonal SP-C (1:50) (#sc13979) (Santa Cruz Biotechnology, USA) or mouse monoclonal antibody to surfactant protein A (SP-A) (1:200) (#ab51891) (Abcam, USA) over night in a moist chamber at 4 °C. For detection Cy3 labelled anti rabbit secondary antibody was used for E-cadherin and SP-C, while a alkaline phosphatase labeling was used to detect SP-A. A549 cells were treated with TN or TG, for 18 hours and 7 days respectively and grown in either BMSC-cm or normal media for 24 hours. Cells were then fixed in 3% PFA as described above and subsequently stained overnight at 4 °C in a moist box with anti-cytokeratin, pan-FITC anti-mouse (1:200) (#C5992), mouse anti-vimentin-Cy3 (1:1000) (both: Sigma, USA) (#C9080) or caspase-3 (1:200) (#9662 S) (Cell Signaling, USA). Samples were then washed 3 times with PBS and analyzed under a confocal microscope LSM 510 Carl Zeiss or under Leica DMI4000D microscope.

### Laser Scanning Microscopy and image restoration

We used Zeiss LSM 710 with an inverted Zeiss microscope (Axiovert 200 M, Lasers: HeNe 633 nm, HeNe 543 nm, and Ar 488 nm) with ×40 objective lens. To improve visualization we processed images with IMARIS 7 software (Bitplane AG, Switzerland). The surpass module was used to localize cells; we performed volume rendering, which displays the volume of the entire data set. Rendering creates an artificial solid object to represent the range of interest of a volume object.

### Quantitative qRT-PCR for determining ER stress markers

To determine ER stress we analyzed the presence of GRP78 (HSPAS (HGNC: 5238)), XBP-1(s) (spliced XBP-1) (XBP1 (HGNC: 12801)) and CHOP (DDIT3 (HGNC: 2726)) and three ER sensors ATF6 (ATF6 (HGNC: 791)), PERK (EIF2AK3 (HGNC: 3255)) and IREIα (ERN1 (HGNC: 3449)), Surfactant protein C (SPC) (UCN3 (HGNC: 17781)) with real time PCR. Primary AEC or ER stressed A549 were treated with different media for 24 hours and Nucleospin RNA extraction kit (Macherey-Nagel, Düren Germany) was used to lyse for RNA extraction. Random hexamers primers were used for the reverse transcriptase (RT) polymerase chain reaction of 1 μg total RNA in a reaction volume of 20 μl; using Omniscript RT kit (Qiagen, USA). Real-time qRT-PCR was performed using the Quantitect Probe PCR kit with SYBER green reagent (Applied Bioscience, USA). The RT PCR detection system (7500 fast real time PCR system, Applied Bioscience) was used with the following RT-cycle conditions: 95 °C for 2 minutes, followed by 40 cycles at 95 °C for 3 seconds, and 60 °C for 30 seconds. For each gene, crossing point (Cp) values were determined from the linear region of the amplification plot. Obtained Cp values were normalized by subtracting the Cp value for 18S mRNA (RNA18S1HGNC:44278) serving as housekeeping gene and used as the base to generate the ΔCp value. The relative change was determined by subtracting the ΔCp value of control samples from the ΔCp value of treated samples, which returned the ΔΔCp value. Primer sequences were synthesized at Microsynth AG (Balgach Switzerland) and can be found in the supplementary data.

### Western Blot

The BioRad protein quantification reagent (Bio-Rad Laboratories, USA) was used to determine total protein concentration. Proteins were resolved by SDS–PAGE, transferred onto nitrocellulose membranes, and incubated with indicated primary antibodies. An ECL kit was used to detect secondary antibodies conjugated to horseradish peroxidase (Amersham Pharmacia Biotech, UK). ECL signals were quantified using Image J software (USA).

### Antibodies for Western blot

Phospho-MET (Tyr1234/35) (#3126), total MET (#4560), and Pathscan multiplex western cocktail I (phospho-p90RSK, phospho-AKT, phospho-p42/44 MAPK, phospho-S6, Rab11) (#5301) Met (D1C2) (#8198), Caspase-3 (#9662) AKT (#9272), eIF2 α (#9722), phospho-eIF2α (Ser51) (#9721), antibodies were purchased from Cell Signaling Technology (Danvers, MA, USA). β-actin (#MAB1501) antibody was from (Millipore Corporation, USA) and ERK 1 (K-23) (#sc-94) from (Santa Cruz Biotechnology, Santa Cruz, USA).

### Proliferation assay

Quick cell proliferation colorimetric assay (Biovision Incorp, USA) was used to quantify cell proliferation, following the protocol provided by the manufacturer. The assay is based on the cleavage of the tetrazolium salt to formazan by cellular mitochondrial dehydrogenase. 1 × 10^4^ cells/well were plated in 96 well plates and treated with TN and TG for 18 hours and 7 days respectively. The cells were then treated with BMSC-cm or BMSC-cm treated with anti HGF antibody for 24 hours. WST was added and the plate was incubated at 37 °C for 4 hours and absorbance was read at 420 nm with reference wave length of 650 nm using Tecan M1000 plate reader (Tecan AG, Switzerland).

### Statistical analysis

Three independent set of experiments were performed for all the experiments, data of one independent experiment is shown. Data are presented as mean ± SEM. We used non-parametric tests, Student’s t-test, or one-way ANOVA, as required, to perform statistical analyses, using GraphPad Prism 5 (GraphPad Software, San Diego, CA) Significance level was p < 0.05.

## Results

### AEC isolated from lung tissue express epithelial markers

**Figure 1:**
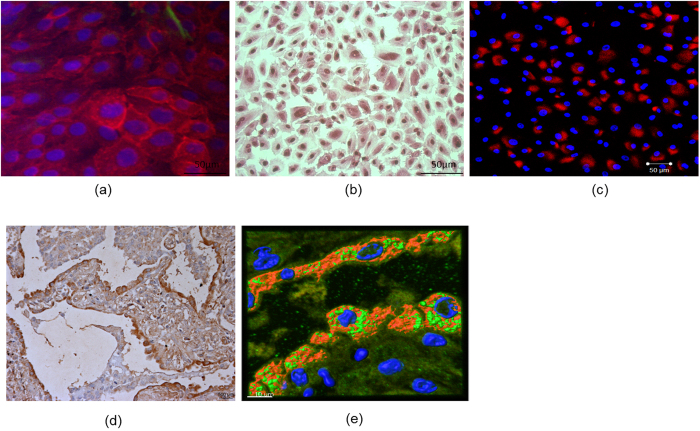
Characterization of isolated AEC was performed by immunostaining. The cells stained for E cadherin performed by immuno-fluroscence (**a**), the cells were also stained for surfactant protein A, positive cells stain pink by alkaline phosphatase staining (**b**). Cells also stained for Surfactant protein C (SP-c) as shown with positive immunoflurosence (**c**). Immunostaining for the ER stress marker GRP78 was performed on the paraffin embedded IPF lung tissue. A distinct staining of the epithelial lining for Grp 78 was seen in area around thickened interstitium. Single mononuclear cell lining the airspaces showed strong staining, based on their location and shape they are likely the alveolar epithelial cells (**d**). Grp78 positive cells (red) are also positive for SPC (green) thus confirming that AEC are under ER stress in IPF patients (**e**). (Image processed by Imaris for volume rendering for clear view) (Scale bar (**a–c**) (50 μm), (**d**) (500 μm), (**e**) (10 μm).

The characterization of the cells isolated from lung biopsies was performed by epithelial marker staining. The cells grew as cobble stone morphology and stained positive for E cadherin (Fig. 1a) expressed Surfactant protein A (SP-A) (Fig. 1b) and Surfactant protein C (SP-C) (Fig. 1c).

### AEC express ER stress marker GRP78 in IPF patients

To evaluate ER stress in IPF lungs, paraffin-embedded lung tissue from IPF patients (n = 3) was subjected to immunohistochemistry for GRP 78 expression. As shown in Fig. 1d, GRP 78 immuno-reactivity was strongly evident in the hyperplastic alveolar epithelium. Cytoplasmic granules in single mononuclear cells lined the thickened interstitium of the lung parenchyma. Cells positive for GRP 78 were alveolar epithelial cells, since immunofluroscence imaging revealed co-localization of GRP 78 and SPC, as shown in Fig. 1e.

### BMSC-cm treatment reduces ER stress in primary AEC isolated from IPF patients

**Figure 2:**
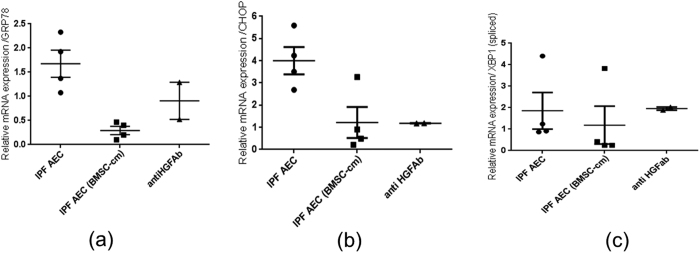
Relative mRNA expression of the ER stress markers measured in primary alveolar epithelial cells isolated from the IPF patients by RT PCR; relative gene expression levels of GRP78 (**a**) CHOP (**b**) and XBP-1 (**c**) normalized to primary alveolar epithelial cells obtained from healthy lungs are demonstrated. After treatment with BMSC-cm the gene expression of GRP78 and CHOP was significantly reduced, however there was no significant change in spliced XBP-1. Moreover, there was no significant increase in the mRNA expression of any ER stress marker after neutralizing HGF in the BMSC-cm. Relative gene expression is represented as ΔΔCT and expressed as mean ± SEM. Experiments were performed in triplicates (n = 3).

The relative mRNA expression levels of ER stress markers GRP78 (1.67 ± 0.28), CHOP (4.0 ± 0.61) and spliced XBP1 (1.85 ± 0.85) were elevated in primary AEC obtained from IPF patients compared to healthy controls. To study the effect of secreted mediators from BMSC-cm, primary AEC were treated with BMSC-cm for 24 hours. After treatment with BMSC-cm the mRNA expression levels were significantly reduced GRP 78 0.29 ± 0.08 vs 1.67 ± 0.28 (p < 0.05) (Fig. 2a) and CHOP (1.2 ± 0.69) vs 4.0 ± 0.61 (p < 0.05) (Fig. 2b). However, the reduction of the spliced XBP-1 level (1.85 ± 0.85) vs 1.18 ± 0.88 was not significant (Fig. 2c). Interestingly, when BMSC-cm was treated with neutralizing HGF antibody there was slight but not significant increase in the ER stress markers; GRP 78 (0.9 ± 0.38), CHOP (1.18 ± 0.03), XBP-1 (s) (1.9 ± 0.07) respectively. Relative mRNA expression was normalized with 18S as housekeeping to primary AEC obtained from healthy lungs.

### BMSC-cm attenuates ER stress in an *in vitro* cell model of ER stress in alveolar epithelial like A549 cells

**Figure 3:**
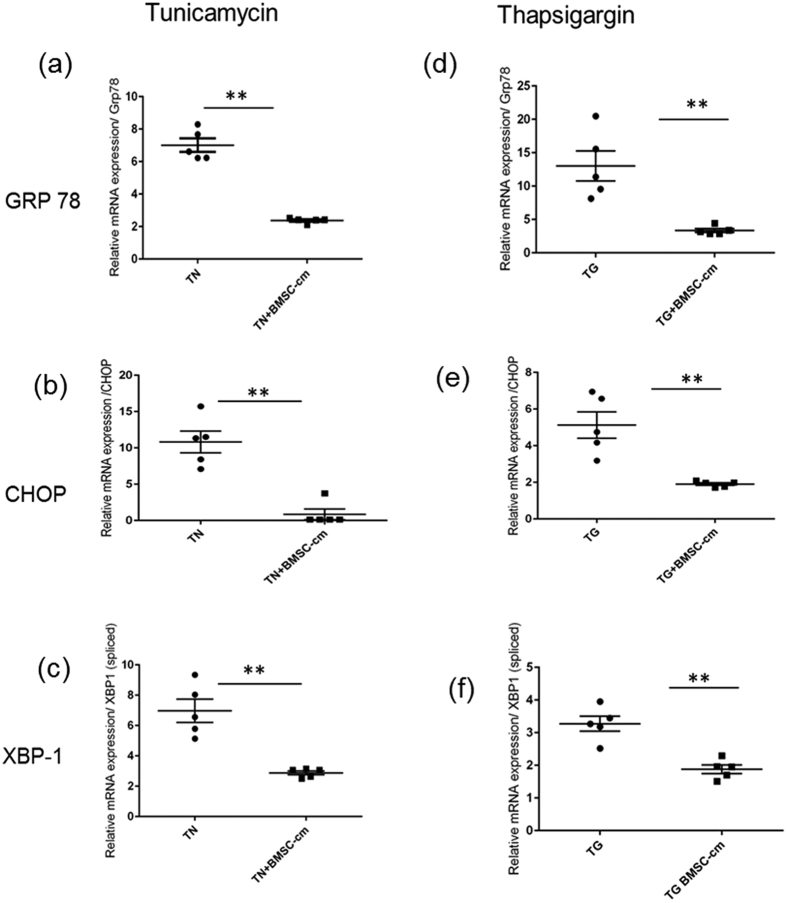
Relative mRNA expression of the markers of ER stress as measured by RT PCR after treatment of ER stressed A 549 cells with BMSC-cm. Gene expression levels of ER stress makers GRP78 (**a,d**) CHOP (**b,e**) and XBP-1 (**c,f**) are significantly reduced. Relative mRNA expression represented as ΔΔCT is expressed as mean ± SEM. Experiments were performed in triplicates (n = 3).

To generate ER stressed lung epithelial cells, A549 cells were grown in monolayer and treated with either TN (2 μg/ml) for 18 hours or TG (0.1 μM) for 7 days. Markers of ER stress were evaluated by qRT-PCR. Relative mRNA expression of ER stress markers relative to untreated A549 cells was significantly upregulated; GRP78 after treatment with TN 7.01 ± 0.41 and 13.02 ± 2.24 after TG, CHOP after TN treatment 10.82 ± 1.49 and 5.12 ± 0.71 after TG treatment, spliced variant of XBP-1(s) after TN treatment 9.41 ± 0.65 and 3.27 ± 0.23 after TG treatment. When TN-induced ER stressed A549 were treated with BMSC-cm for 24 hours relative mRNA expression of GRP 78 decreased to 2.46 ± 0.08 vs. 7.01 ± 0.41 (p < 0.001) (Fig. 3a). Relative mRNA expression levels for CHOP were similarly reduced 3.22 ± 0.68 vs. 10.82 ± 1.49 (p < 0.001 (Fig. 3b), and spliced XBP1 were also reduced 4.05 ± 0.28 vs. 9.41 ± 0.65 (p < 0.001) (Fig. 3c) compared to untreated ER stressed A549 cells. When TG induced ER stressed A549 were treated with BMSC-cm for 24 hours, the mRNA expression level of all the markers also significantly reduced; GRP78 3.82 ± 0.533 vs. 13.02 ± 2.24 (p < 0.001) (Fig. 3d); CHOP 2.15 ± 0.26 vs. 5.12 ± 0.71 (p < 0.005) (Fig. 3e); and, spliced XBP-1 1.98 ± 0.15 vs. 3.27 ± 0.23 (p < 0.0005) (Fig. 3d). All values are expressed as mean ± SEM and represented as ΔΔCT relative mRNA expression, with 18 S as a housekeeping gene normalized to untreated A549 cells.

### ER stress induced epithelial-to-mesenchymal transition (EMT) is reversed after BMSC-cm treatment

**Figure 4:**
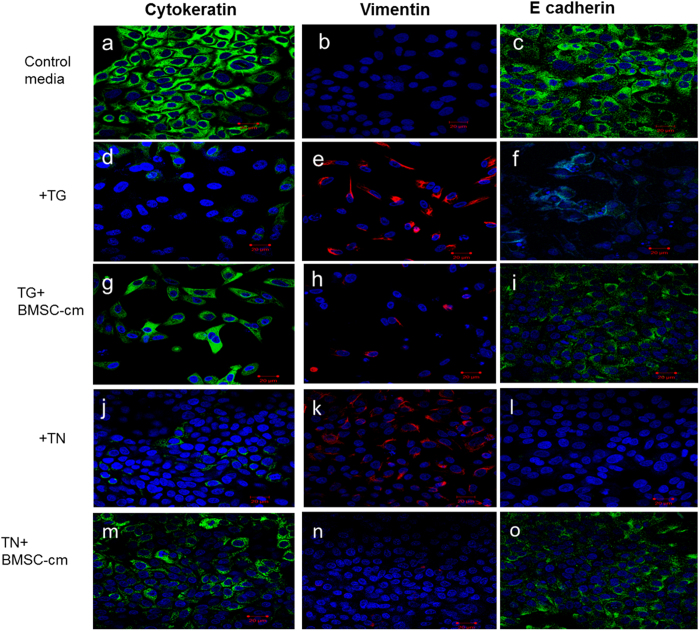
After induction with TN or TG the A549 cells lost the typical cobble stone morphology of alveolar epithelial cells, and changed to elongated fibroblast like morphology. Additionally these cells lost cytokeratin (**d,j**) and E cadherin (**f**,**l**) expression but expressed vimentin (**e,k**). Treatment with BMSC-cm helped restore the morphology of the ER stressed A549 cells, back to that of the epithelial cells, the expression of vimentin was lost (**h,n**) cytokeratin (**g,m**) and E cadherin (**i,o**) expressions were restored. (Scale bar 20 μm for all figures). Experiments were performed in triplicates (n = 3).

After treatment with TN or TG, the epithelial cobblestone morphology of A549 cells changed to an elongated spindle-like shape typical of mesenchymal cells like fibroblasts. This phenotype change of AEC into a mesenchymal cell type was confirmed by positive immuno-fluroscence staining of vimentin (Fig. 4e,k) and weak staining for epithelial markers cytokeratin (Fig. 4d,j) and E cadherin (Fig. 4f,l). After incubating with BMSC-cm for 24 hours, the TN or TG induced changes in ER-stressed AEC were restored. The elongated fibroblast like appearance changed into cobblestone epithelial cells, and vimentin expression was not observed, (Fig. 4h,n) while confocal microscopy confirmed that cytokeratin (Fig. 4g,m) and E cadherin (Fig. 4i,o) expression was restored.

### BMSC-cm treatment improves alveolar epithelial repair capacity of ER stressed A549 cells *in vitro*

**Figure 5:**
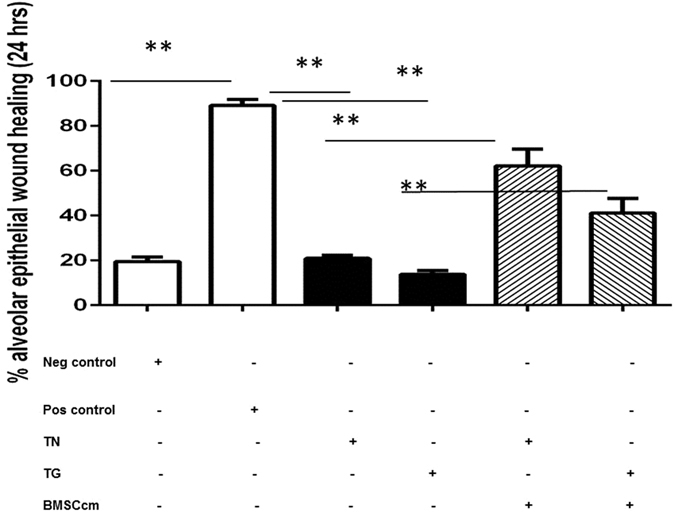
*In vitro* alveolar epithelial wound repair after 24 hours was impaired, in both TN and TG stressed A 549 cells, compared to controls. Data is shown as % of alveolar epithelial wound healing and values are expressed as mean ± SEM. Experiments were performed in triplicates (n = 3).

A wound was mechanically created in a confluent monolayer of A549 cells in presence or absence of ER stress-inducing factors (TN, TG). Wound healing response in ER-stressed cells was drastically reduced after 24 hours, 20.9 ± 1.3% in TN treated and 13.8 ± 1.6% in TG treated A549 cells (p < 0.0001), compared to 89.1 ± 2.7% alveolar epithelial wound repair in cells growing in 10% FCS (positive control), and 19.5 ± 1.9% wound closure in cells growing in serum-free media (negative control). ER stressed A549 cells were treated with BMSC-cm and alveolar epithelial wound repair was assessed 24 hours later. Significant alveolar epithelial wound repair was observed in TN induced cells after BMSC-cm treatment (53.4 ± 3.3% vs. 20.9 ± 1.3% (p < 0.001)). Similarly, alveolar epithelial wound healing was markedly improved when TG induced ER-stressed A549 cells were treated with BMSC-cm (46.6 ± 3.5% vs. 12.0 ± 1.8% wound repair in non-treated cells (p < 0.01)), (Fig. 5). Results are shown as percentage of wound surface area closure after 24 hours; values are expressed as mean ± SEM.

### ER stress reliving action of BMSC-cm is partially mediated by HGF

Biologically relevant levels of HGF were detected in the BMSC-cm. HGF levels in BMSC-cm were 224.3 ± 6.67 pg/ml.

To study if HGF mediated the beneficial effect of BMSC-cm on stressed AEC, HGF in BMSC-cm was inhibited by pretreatment with anti-HGF antibody (0.8 ng/ml) for 7 minutes at 37 °C.

**Figure 6:**
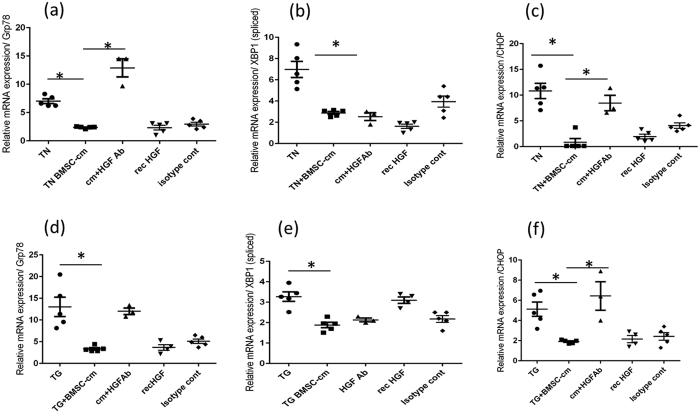
BMSC-cm was treated with anti HGF antibody to neutralize HGF. The relative mRNA expression of Grp78 (**a,d**) and CHOP (**b,e**) was increased after treatment of ER stressed A 549 cells with HGF neutralized BMSC-cm, however XBP1-(s) (**c,f**) levels were not affected. Similarly, the relative mRNA expression of Grp 78, CHOP and XBP-1 (s) was reduced after recombinant HGF treatment in TN stressed A 549 cells. In the TG stressed A 549 cells the mRNA expression was reduced for GRP78 and CHOP, however, XBP-1 (s) showed increase after recombinant HGF treatment. BMSC-cm when treated with isotype control antibody had no change in relative mRNA expression. Relative mRNA expression represented as ΔΔCT is expressed as mean ± SEM. Experiments were performed in triplicates (n = 3).

ER-stressed A549 were treated with HGF neutralized BMSC-cm for 24 hours, relative mRNA expression of two ER stress markers significantly increased again GRP78 (12.90 ± 1.60) p < 0.0001) (Fig. 6a) and CHOP (8.46 ± 1.48) (p < 0.0001) (Fig. 6b). The expression of spliced XBP-1 was not influenced by HGF neutralization (2.53 ± 0.37); (Fig. 6c). Results were similar when TG-stressed A 549 were treated with HGF neutralized BMSC-cm; expression levels again increased for GRP78 (12.00 ± 0.75) (p < 0.001) (Fig. 6d), CHOP (6.43 ± 1.41) (p < 0.001) (Fig. 6e), but not for spliced XBP-1 (2.13 ± 0.08) (Fig. 6f). To study the effect of HGF on ER stress, ER stressed A549 cells were treated with recombinant HGF (1.6 ng/ml). Interestingly, the level of ER stress markers decreased in TN treated A549 cells the mRNA expression of GRP78 (2.32 ± 0.41), CHOP (1.95 ± 0.44), and spliced XBP-1 (1.62 ± 0.19) was observed. Similarly, in TG stressed A549 cells reduction in GRP 78 (3.67 ± 0.64) and CHOP (2.15 ± 0.35) was observed. However the mRNA expression of spliced XBP-1 showed a slight increase after HGF treatment (3.10 ± 0.15).

Additional control was performed where BMSC-cm was treated with isotype antibody and mRNA expression was measured with similar results obtained in the BMSC-cm treated group. When TN stressed A549 cells were incubated with isotype antibody ER stress markers were in the same range as in the BMSC-cm group: GRP78 (2.94 ± 0.33), XBP-1 (3.9 ± 0.52) and CHOP (4.07 ± 0.52). Similarly, in TG stressed A549 cells, mRNA expression was: GRP 78 (5.1 ± 0.51) XBP-1 (2.18 ± 0.16) and CHOP (2.42 ± 0.37) (Fig. 6).

### *In vitro* alveolar epithelial wound repair is mediated by HGF present in the BMSC-cm

**Figure 7:**
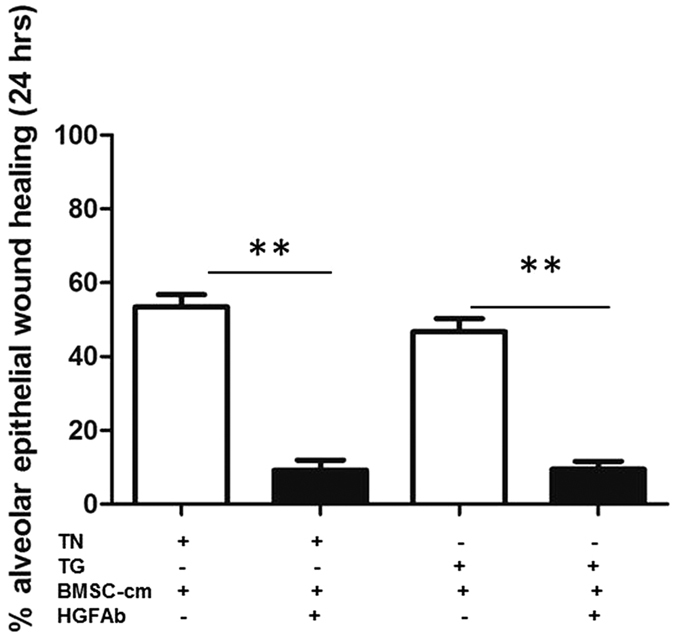
Alveolar epithelial wound repair invitro was markedly reduced after HGF neutralized BMSC-cm. Data is represented as % of alveolar epithelial wound healing and values are expressed as Mean ± SEM. Experiments were performed in triplicates (n = 3).

To uncover the role of HGF in alveolar epithelial wound repair in ER stressed cells, BMSC-cm was treated with HGF neutralizing antibody and wound-healing was assayed on TN or TG stressed A549 cells. Wound healing capacity was severely reduced after HGF was neutralized in BMSC-cm 9.1 ± 2.7% in TN treated cells and 9.4 ± 2.1% in TG treated cells after 24 hours (p < 0.001) (Fig. 7).

### BMSC-cm restores unfolded protein response (UPR) homeostasis and mediated by HGF

**Figure 8:**
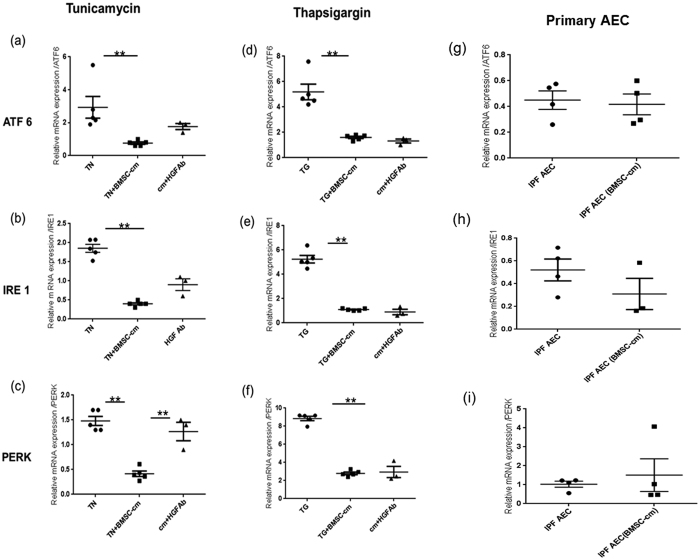
BMSC-cm treatment reduced the expression of three transmembrane protein sensors, ATF6 (**a,d**), IRE1 (**b,e**) and PERK (**c,f**) in TN and TG treated A 549 cells. mRNA expression levels of and ATF6, IRE1 were slightly increased after neutralization of HGF in BMSC-cm, whereas PERK levels were significantly increased in TN stressed A549 cells. However, no change was observed in the mRNA expression levels of PERK, ATF6, IRE1 in TG stressed A 549 cells. Moreover, no change in the mRNA expression levels of three UPR sensors in primary AEC cells obtained from IPF patients (**g**, **h**, and **i**). Experiments were performed in triplicates (n = 3).

UPR is signaled via three trans-membrane protein sensors: ATF6, IRE1, and PERK. In TN-induced ER-stressed A549 cells, relative mRNA expression of ATF6 (2.93 ± 0.65), IRE1 (1.85 ± 0.10) and PERK (1.48 ± 0.09) increased. 24 hours after BMSC-cm treatment, the relative mRNA expression of ATF6 (1.03 ± 0.03) (p < 0.01), IRE1 (0.43 ± 0.03) (p < 0.01) and PERK (0.41 ± 0.05) (p < 0.01) significantly decreased (Fig. 8(a–c). Similarly, in A549 cells treated with TG, higher relative mRNA expression levels of ATF6 (5.19 ± 0.61), IRE1 (5.24 ± 0.31) and PERK (8.8 ± 0.23) was observed. After treatment with BMSC-cm, relative mRNA expression dropped to (2.79 ± 0.1) PERK (p < 0.001), (1.10 ± 0.02) IRE1 (p < 0.0001), and (1.60 ± 0.09) ATF6 (p < 0.001) (Fig. 8(d–f). Interestingly, we detected no change in the mRNA expression pattern of any sensors in the IPF AEC obtained from patients Fig. (8g–i). Moreover, mRNA expression of two protein sensors ATF 6 (1.76 ± 0.18) (p < 0.01) (Fig. 8a) and PERK (1.26 ± 0.18) (p < 0.01) (Fig. 8c) significantly increased after treatment with HGF-neutralized BMSC-cm, but IRE1 (0.90 ± 0.15) (Fig. 8b) did not show any significant change in the TN stressed A 549 cells. In contrast, treating TG stressed A549 cells with HGF-neutralized BMSC-cm had no effect on the mRNA expression levels of any of the three sensors: PERK (2.94 ± 0.61), ATF 6 (1.3 ± 0.15) and IRE1 (0.90 ± 0.22) (Fig. 8d,e). The values are expressed as mean ± SEM and represented as ΔΔCT relative gene expression; 18S is the housekeeping gene normalized to normal A549 cells.

### hSPC mRNA expression in the AEC is partially restored after BMSC-cm treatment

To analyze the effect of HGF on alveolar homeostasis, we analyzed surfactant protein C expression on AEC obtained from IPF patients. hSPC mRNA expression in normal AEC (obtained from non-fibrotic lung) was 1.43 ± 0.01, however mRNA expression of hSPC increased after BMSC-cm treatment (4.49 ± 0.84) compared to the untreated IPF AEC (1.62 ± 0.17) (p < 0.001). Gene expression was reduced when BMSC-cm was treated with anti-HGFAb (0.40 ± 0.21) (p < 0.01) (Supplementary Fig. S1). Values are expressed as mean ± SEM and represented as ΔΔCT relative mRNA expression normalized to lung fibroblasts.

### HGF present in BMSC-cm acts via c-MET receptor signaling

We performed western blot to study the activation of the HGF receptor, the c-MET receptor tyrosine kinase, and how HGF signals downstream of c-MET after BMSC-cm treatment. Autophosphorylation levels of c-MET (Tyr1234/1235) and phosphorylation of AKT (Ser473), and ERK1/2 (Thr202/204) all increased in ER-stressed A549 cells after treatment with BMSC-cm. These increased phosphorylation indicates that the MET receptor and subsequently also both the PI3K/AKT/mTOR and MAPK/ERK signaling pathways were concomitantly activated. This effect was more pronounced in TG-induced cells when treated with BMSC-cm as compared to TN-induced BMSC-cm-treated A549 cells (Supplementary Fig. S2a,b).

Total MET protein levels was increased after BMSC-cm treatment and were reduced in presence of HGF neutralizing antibody in both TN- and TG-stressed cells. Total AKT showed a slight increase after BMSC-cm treatment in TN-stressed cells only, whereas ERK 1 showed a decrease after treatment with BMSC-cm in both types of stressed cells.

#### HGF present in the BMSC-cm activates PERK downstream signaling

To further understand the possible downstream mechanisms, we evaluated the expression of translational initiator eIF2α and phospho-eIF2α. After TN- and TG-induced stress, eIF2α was increased; however treatment with BMSC-cm led to reduced eIF2α protein levels. Similarly, increased eIF2α phosphorylation was observed in TN- and TG- treated A549 cells. After BMSC-cm treatment, the phosphorylation levels of eIF2α were reduced and, interestingly, phosphorylation increased again when BMSC-cm was treated with HGF neutralizing antibody. To study the impact of these perturbations on cellular apoptosis, we further assessed the levels of cleaved caspase-3 in A549 cells, which were decreased after BMSC-cm treatment in ER-stressed A549 cells. Cleaved caspase-3 product was detected in TN- and TG-stressed A549 cells, whereas no caspase-3 cleavage was observed after BMSC-cm treatment (Supplementry Fig. S2c,d).

### BMSC-cm treatment increases cell proliferation

To study the effect of c-MET activation after BMSC-cm, cell proliferation assay was performed. Cell proliferation in the normal A549 cells was (1.78 ± 0.116), but was reduced in ER stressed A 549 cells (1.36 ± 0.1) (p < 0.01) in TN treated cells and (1.02 ± 0.05) (p < 0.001) in TG treated group. However, treatment with BMSC-cm for 24 hours improved cell proliferation 2.10 ± 0.05 in TN stressed cells (p < 0.0001) and 1.58 ± 0.08 in TG stressed cells (p < 0.0001). When HGF was neutralized in BMSC-cm, cell proliferation reduced again to 1.33 ± 0.04 in TN stressed cells, (p < 0.001) and 1.17 ± 0.021 in TG stressed cells (p < 0.0001) (Supplementary Fig. S3) optical density is presented as mean ± SEM.

### TGF-β_1_ treatment does not induce ER stress response neither ER stress induction increases TGF-β1 levels

To understand the cause-effect relationship between fibrosis and ER stress, we treated A549 cells with 8 ng/ml recombinant TGF-β_1_ and measured the mRNA expression levels of GRP78, spliced XBP-1, and CHOP 6 hours, 24 hours and 48 hours after TGF-β_1_ treatment. None of the three markers significantly changed at any point after TGF-β_1_ treatment. GRP78 mRNA expression was 0.68 ± 0.05 6 hours; 0.93 ± 0.28 24 hours and 0.94 ± 0.17 48 hours after TGF β_1_ treatment. Similarly, spliced XBP1 expression after 6 hours 0.51 ± 0.03, 1.08 ± 0.29 at 24 hours and 0.87 ± 0.04 after 48 hours, and CHOP expression at 6 hour 0.68 ± 0.05, at 24 hour 0.93 ± 0.28 and at 48 hour 0.94 ± 0.17 did not show any significant increase. Furthermore, A549 cells treated with TN or TG did not show any change in TGF- β1 levels TN (1 ± 0.14) and TG (1.05 ± 0.13) (Supplementary Fig. S6). Values represented as ΔΔCT relative mRNA expression, and expressed as mean ± SEM.

## Discussion

In this study we show that AEC from IPF patients are under ER stress. Biological activities relevant in repair and restoration such as alveolar epithelial repair and proliferation are impaired under ER stress. BMSC-cm treatment reduced ER stress, increased cell proliferation and improved wound healing in alveolar epithelial cells. We demonstrate that these effects are partially mediated by HGF present in BMSC-cm; and therefore suggest BMSC-cm as a potential novel therapeutic option for repair and regeneration in fibrotic lungs.

Endoplasmic reticulum (ER) is an intracellular organelle where secreted and membrane proteins are properly folded, calcium homeostasis is regulated, and lipids and steroids are produced^26^. Any event disturbing the ER homeostasis leads to ER stress. Few pulmonary diseases including pulmonary fibrosis have been associated with increased ER stress^27^,^28^. We observed GRP78 positive cells in the lung which co-stained with SPC and identified them as AECII like other researchers^5^. Gene expression levels of GRP78, XBP-1(s) and CHOP in isolated primary AEC from IPF patients were elevated, confirming that AEC in fibrotic lungs are under ER stress. In our study, *in vitro* alveolar epithelial repair was markedly impaired in ER stressed alveolar epithelial cells, suggesting that ER stress may play important active role in improper alveolar epithelial repair, and thus may contribute to the pathophysiology of pulmonary fibrosis.

Various disease models and some clinical trials have shown the regenerative potential of bone marrow stromal cells (BMSC)^29^,^30^. Recent studies demonstrated that applying conditioned media of stem cells improved tissue repair and regeneration in several disease models^31^,^32^,^33^. We found that BMSC-cm treatment reduced ER stress and significantly and improved alveolar epithelial repair capacity of ER stressed alveolar epithelial cells *in vitro*.

The conditioned media obtained from BMSC is rich in various growth factors. In previous studies we demonstrated the anti-fibrotic role of HGF in animal models of lung fibrosis, using either gene therapy^34^,^35^ or BMSC over expressing HGF^16^. We and others have elaborated several anti-fibrotic mechanisms of HGF^36^,^37^. However, the effect of BMSC-cm and its contents on ER homeostasis is still unknown.

It is hypothesized that fibroblasts of IPF patients do not produce enough HGF to support re-epithelialization of alveolar epithelium after injury^38^. We observed wound healing on ER stressed A549 monolayer was faster in presence of BMSC-cm. Neutralizing HGF in the BMSC-cm significantly reduced alveolar epithelial repair. Moreover, blocking HGF increased ER stress markers GRP78 and CHOP significantly in both TN and TG stressed A549 cells, but the expression of spliced variant XBP-1 (s) was unaffected. The role played by HGF may be partially explained by the fact that TN induces ER stress by inhibiting protein N-linked glycosylation which is essential for c-MET function^39^, causes cell cycle arrest in the G1-phase leading to accumulation of unfolded proteins. Thapsigargin (TG) raises cytosolic calcium, and blocks its reuptake in the ER lumen, thereby disrupting protein folding, inducing ER stress^40^ and down regulating c-MET expression^41^. Based on our data we speculate that HGF reverses the TN and TG induced inhibition of c-MET by facilitating N glycosylation and upregulating c-MET signaling, thus attenuating ER stress. PI3K/AKT and MAPK/ERK pathway activation is mediated via CHOP and induces ER stress^42^. HGF is a pleotropic growth factor and potent mitogen^43^ that elicits its anti-apoptotic and proliferative effect via the PI3K/AKT and MAPK/ERK pathways^44^. In the current study we also demonstrate increased expression of total and p-MET, p-AKT, p-ERK, increased proliferation and reduced caspase-3 and cleaved caspase-3 after BMSC-cm treatment of TN and TG stressed A549 cells. Interestingly, when HGF was neutralized in BMSC-cm a significant decrease in p-MET, p-AKT, p-ERK and significant increase in GRP78 and CHOP mRNA was observed in TN and TG stressed A549 cells. Moreover, cell proliferation was significantly reduced and caspase-3 was increased further confirming the essential role of HGF present in the conditioned media.

Furthermore, in the *in vitro* ER stress model TN and TG stressed A549 cells changed phenotype, lost cytokeratin expression, and expressed vimentin, indicating that epithelial cells transform to mesenchymal cells under ER stress. Epithelial to mesenchymal transition (EMT) is an essential process in organogenesis, but its role in IPF is debated. Opinions based on *in vitro* and *in vivo* data differ strongly^45^. *In vitro* EMT has been induced by TN in primary rat alveolar epithelial cells and in A549 cells^46^. In our study, TN and TG induced EMT reversed after being treated with BMSC-cm.

HGF plays an important role in EMT^47^; we have showed earlier that HGF over-expression modulated the process of EMT *in vivo* and is mediated via TGF-β_1_^35^. The role of TGF-β_1_ in ER stress was not established in our study, since ER stress led neither to increased TGF- β_1_ expression, nor did TGF-β_1_ treatment raised ER stress markers. Our results indicate a possible TGF-β_1_ independent pathway as suggested before^9^. Another study found TGF-β_1_ mediated ROS production and induction of ER stress leading to induced fibroblast to myo-fibroblast differentiation^48^, indicating towards a cell-specific relation between TGF-β_1_ and ER stress. Interestingly, however we could only identify cells co-expressing GRP78 and SPC, indicating that only the AEC are under ER stress in IPF.

A further aspect of ER stress is the unfolded protein response (UPR), BMSC-cm treatment restored UPR homeostasis in TN and TG stressed A549 cells by reducing the mRNA expression of all three ER resident transmembrane proteins PERK, IRE1 and ATF6 that signal downstream pathway after sensing ER stress. However, after neutralizing HGF in BMSC-cm, PERK levels significantly increased, but no alteration was observed in IRE1 and ATF6 mRNA expression. In line with these findings decrease in elf2α and p-elf2α were also observed after treatment of BMSC-cm, whereas XBP-1 (s) and ATF6 levels did not show any change, indicating that HGF acts only via regulation of PERK and not involving IRE1 and ATF6. PERK kinase upon activation phosphorylates eIF2α, which is critical in ER stress response and plays essential role in protein synthesis. Phosphorylation of eIF2α leads to downregulation of protein synthesis and inhibition of cyclin D1 translation contributing to cell-cycle arrest. Another function of phosphorylated eIF2α is to activate CHOP, which promotes apoptosis^49^. Interestingly, our data suggests that HGF mediates dephoyphosphorylation of eIF2α, which leads to reduced CHOP levels and increased proliferation in TN stressed AEC. Two other transmembrane protein IRE1 and ATF6 were also analyzed. It is known that IRE1 activation leads to splicing of XBP-1 that triggers upregulation of target genes. However, we did not observe any influence of HGF/c-MET excess on XBP-1 in alignment with previous report^50^. ATF6 activation leads to regulated intramembrane proteolysis,we could not observe any interaction between ATF6 and HGF, and there are no reports available to best of our knowledge describing any possible association.

More interestingly, no effect was observed on any of the three ER resident transmembrane proteins in TG stressed cells. These findings point towards a different mode of action of HGF in attenuating ER stress, depending on the pathway of induction; this however still needs to be investigated. Furthermore, recombinant HGF treatment reduced ER stress markers in TN treated A549 cells, however in TG stressed cells GRP78 and CHOP levels were reduced but the XBP-1 (s) showed no significant change. Also caspase-3 and cleaved caspase-3 was increased in TN stressed A549 cells, and no expression was observed in TG stressed A549 cells, confirming the previous report that TG induced apoptosis is caspase-3 independent^51^. However, after treatment with BMSC-cm, caspase-3 expression was diminished in TN induced ER stressed cells, indicating anti-apoptotic property of BMSC-cm. Furthermore, after neutralizing HGF in the BMSC-cm lead to increase in cleaved caspase indicating possible role of HGF. Based on our observations we state that HGF exerts its anti-apoptotic activity by PI3-kinase/AKT pathway in both TN and TG stressed cells consistent with previous report^44^, however, in TN stressed cells it involves two mechanisms PI3-kinase/AKT and caspase-3. Moreover, these interesting observations need further investigation, importantly what role HGF plays in calcium homeostasis, as it was reported to increase [Ca2+]i levels^52^.

Notably, we observed that restoring ER homeostasis in IPF AEC after BMSC-cm treatment increased the synthesis of surfactant protein C. HGF is a potent mitogen for alveolar epithelial type II cells^53^ and SPC is exclusively produced by alveolar epithelial type II cells. Moreover, SPC mutations play an essential role in development of interstitial lung diseases^54^, which are thought to be mediated via ER stress mechanisms^55^. In our study we demonstrate that upon treatment with BMSC-cm the ER homeostasis was restored leading to increased SPC production which was in part regulated by HGF. However, the exact mechanisms which lead to this novel finding needs to be investigated in more details. We speculate that restoration of ER homeostasis leads to reduced apoptosis, increased cell survival and thus to increased production of SPC. In the current study we were limited in obtaining more patient derived primary AEC, also the BMSC-cm which was tested was from one donor only. However, as a proof of concept we show that BMSC-cm improved alveolar epithelial wound healing, reduces ER stress and reversed EMT in stressed A549 cells, and in primary AEC in patients with IPF. We reaffirm that ER stress plays an important role in pathology of lung fibrosis, and could be an interesting target for treatment of lung fibrosis. We also demonstrated that BMSC-cm attenuated ER stress in part by HGF. To further elaborate on the other mediators in the BMSC-cm, a detailed systems biology approach is warranted, to help understand more precise mechanisms governing the beneficial effects of the secreted mediators. Our findings suggest that BMSC secreted mediators may be used as pharmaco-therapeutics in future to restore ER homeostasis and improve alveolar epithelial repair and regeneration, a novel and promising new approach for lung repair and regeneration in fibrotic lungs.

## Additional Information

**How to cite this article:** Nita, I. *et al*. Hepatocyte growth factor secreted by bone marrow stem cell reduce ER stress and improves repair in alveolar epithelial II cells. *Sci. Rep.* **7**, 41901; doi: 10.1038/srep41901 (2017).

**Publisher's note:** Springer Nature remains neutral with regard to jurisdictional claims in published maps and institutional affiliations.

## Supplementary Material

Supplementary Data
