# Supplementary material for: Hepatocyte growth factor secreted by bone marrow stem cell reduce ER stress and improves repair in alveolar epithelial II cells
Source: Sci Rep. 2017 Feb 3;7:41901. doi: 10.1038/srep41901 (PMC5291222; doi:10.1038/srep41901)
Supplement: Supplementary Data [file srep41901-s1.pdf]

Supplementary File

Hepatocyte growth factor secreted by bone marrow stem cell reduce ER stress and improves repair in alveolar epithelial II cells

Izabela Nita[1,2], Katrin Hostettler[3],Luca Tamo[1,2,4], Michaela Medová [2,5], Giuseppe Bombaci,[2,4,6] Jun Zhong[3], Ramanjaneyulu Allam [2,6], Yitzhak Zimmer [2,5], Michael Roth[3], Thomas Geiser[1,2*] , Amiq Gazdhar[1,2*]

1) Department of Pulmonary Medicine, University Hospital Bern, Switzerland. 2) Department of Clinical Research University of Bern, Switzerland. 3) Pulmonary Cell Research, Department of Biomedicine, University and University Hospital Basel, Basel, Switzerland. 4) Graduate school of Biomedical science, University of Bern, Bern Switzerland. 5) Department of Radiation Oncology, Inselspital, Bern University Hospital, and University of Bern, Bern, Switzerland. 6) Department of Hematology, University Hospital Bern, Bern, Switzerland.

Supp Fig 1

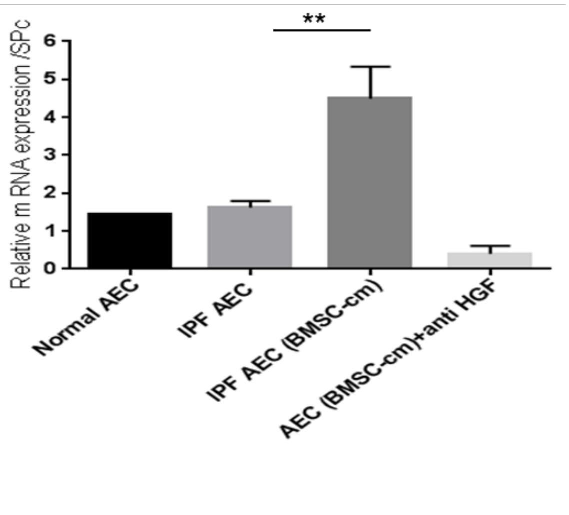

Supp Fig 2 a

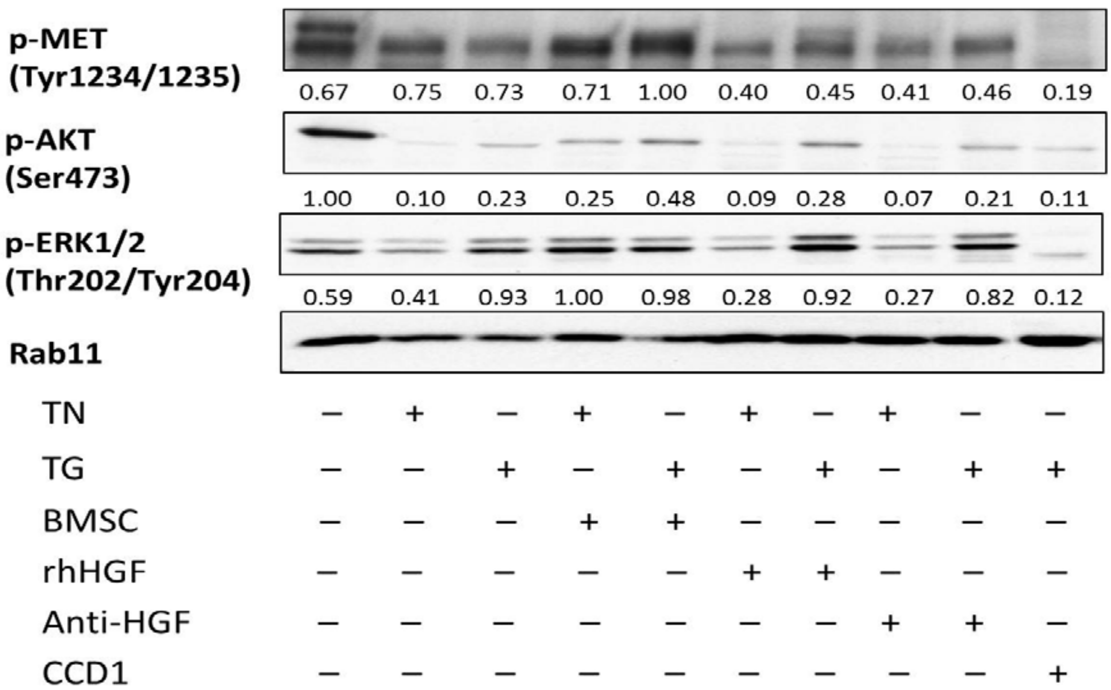

Suppl Fig 2b

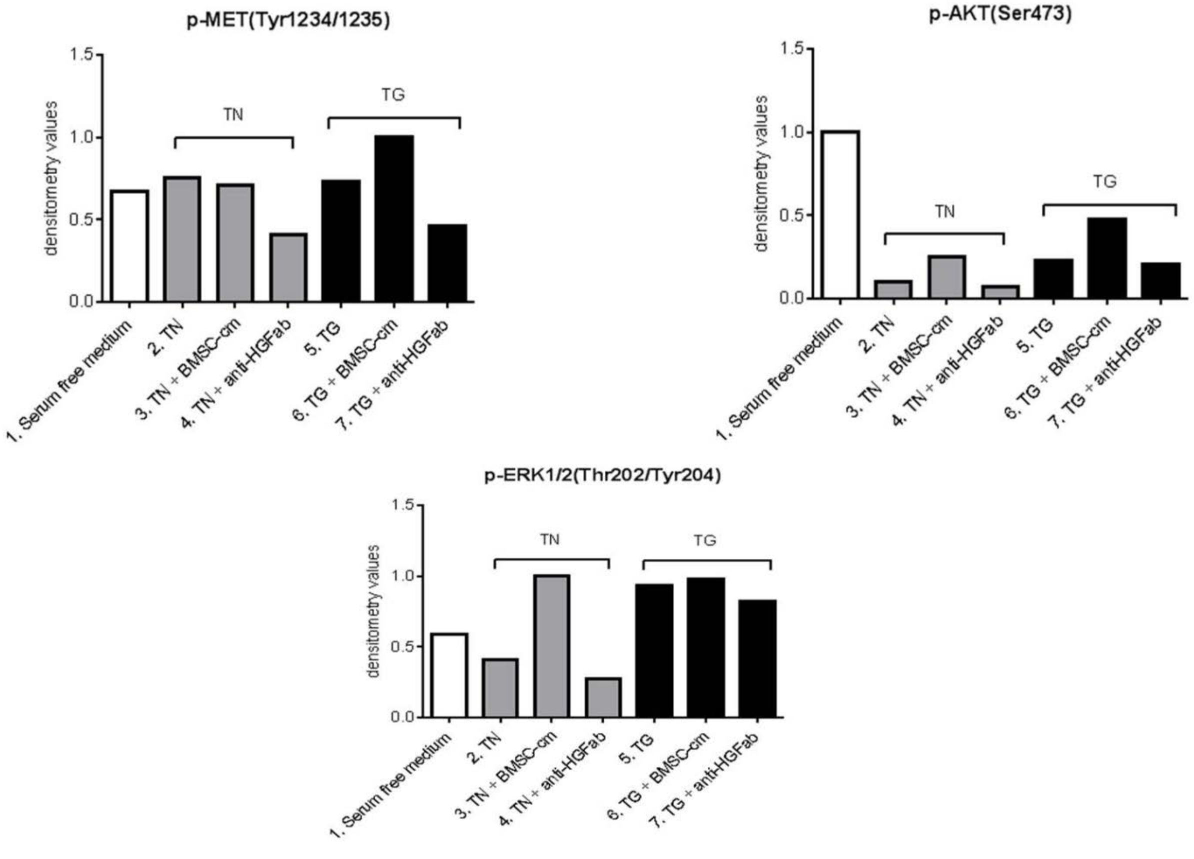

Supp Fig 2c

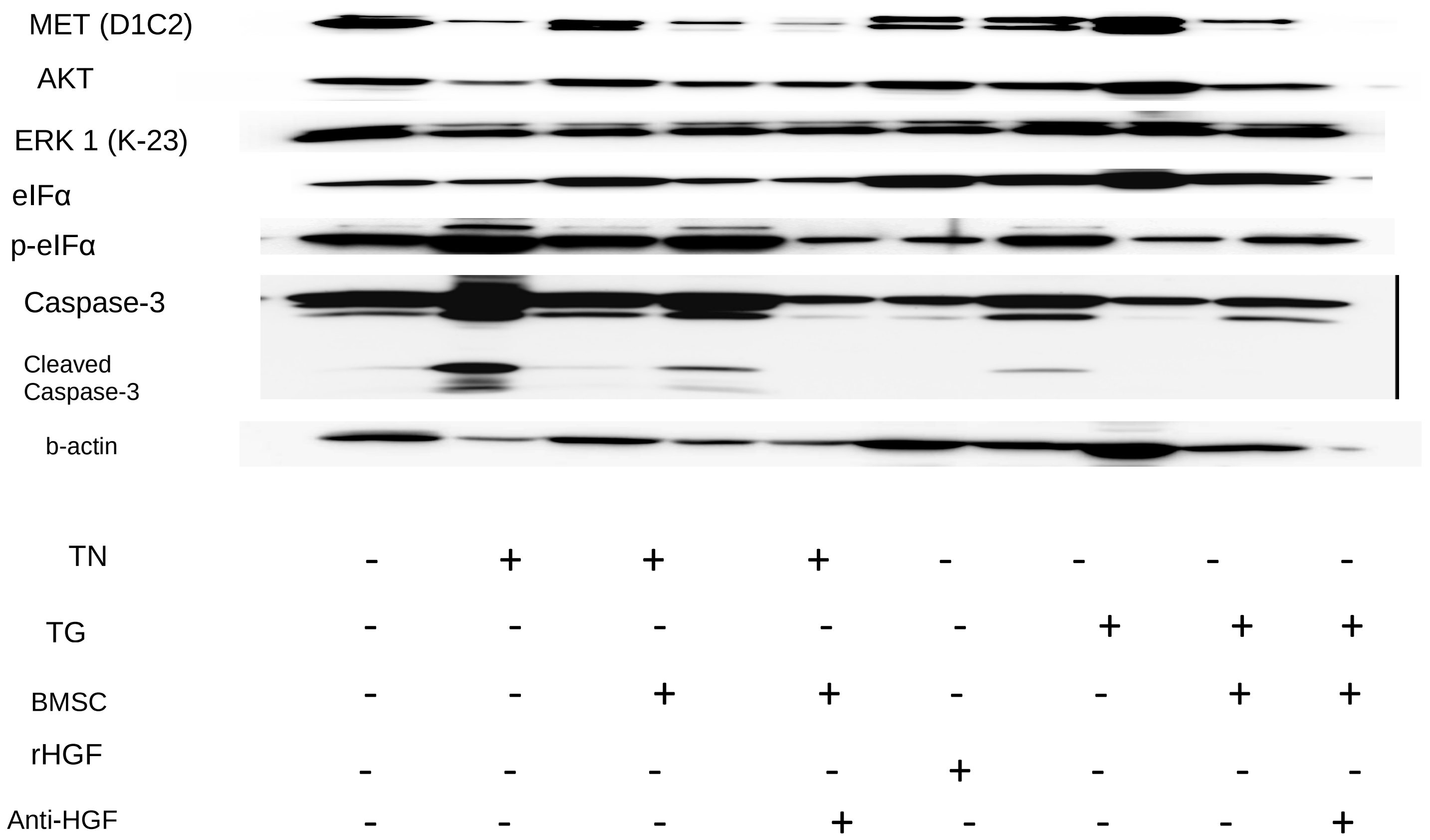

Supp Fig 2d

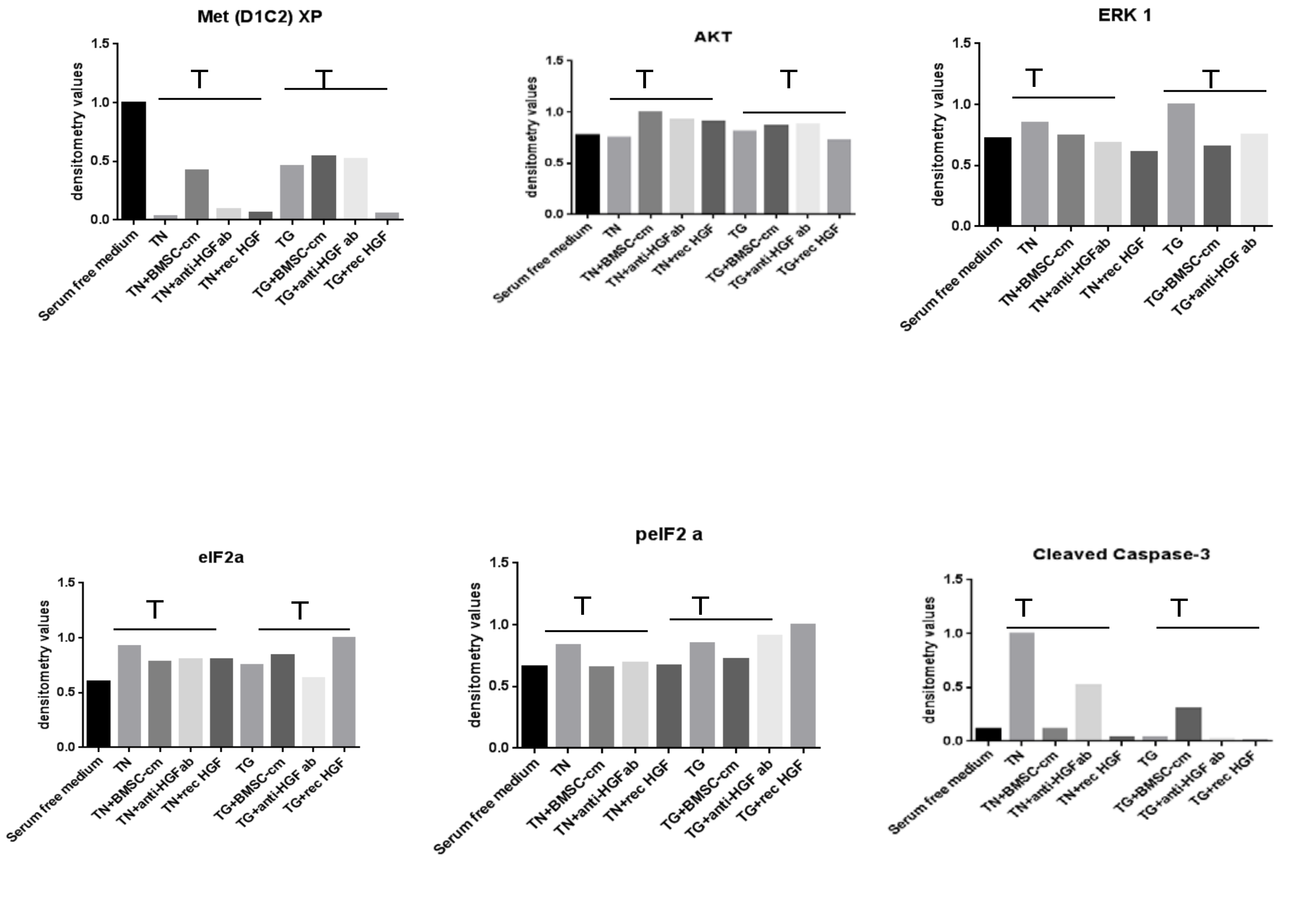

Supp Fig 3

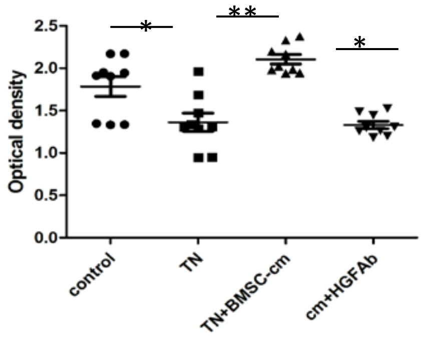

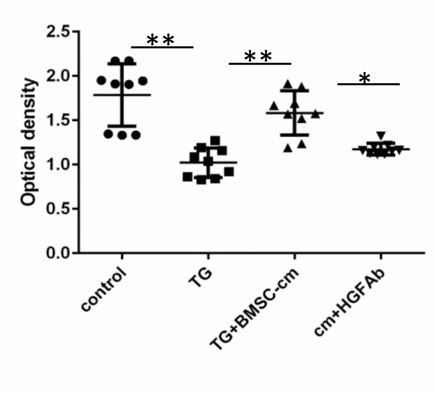

Supp Fig 4

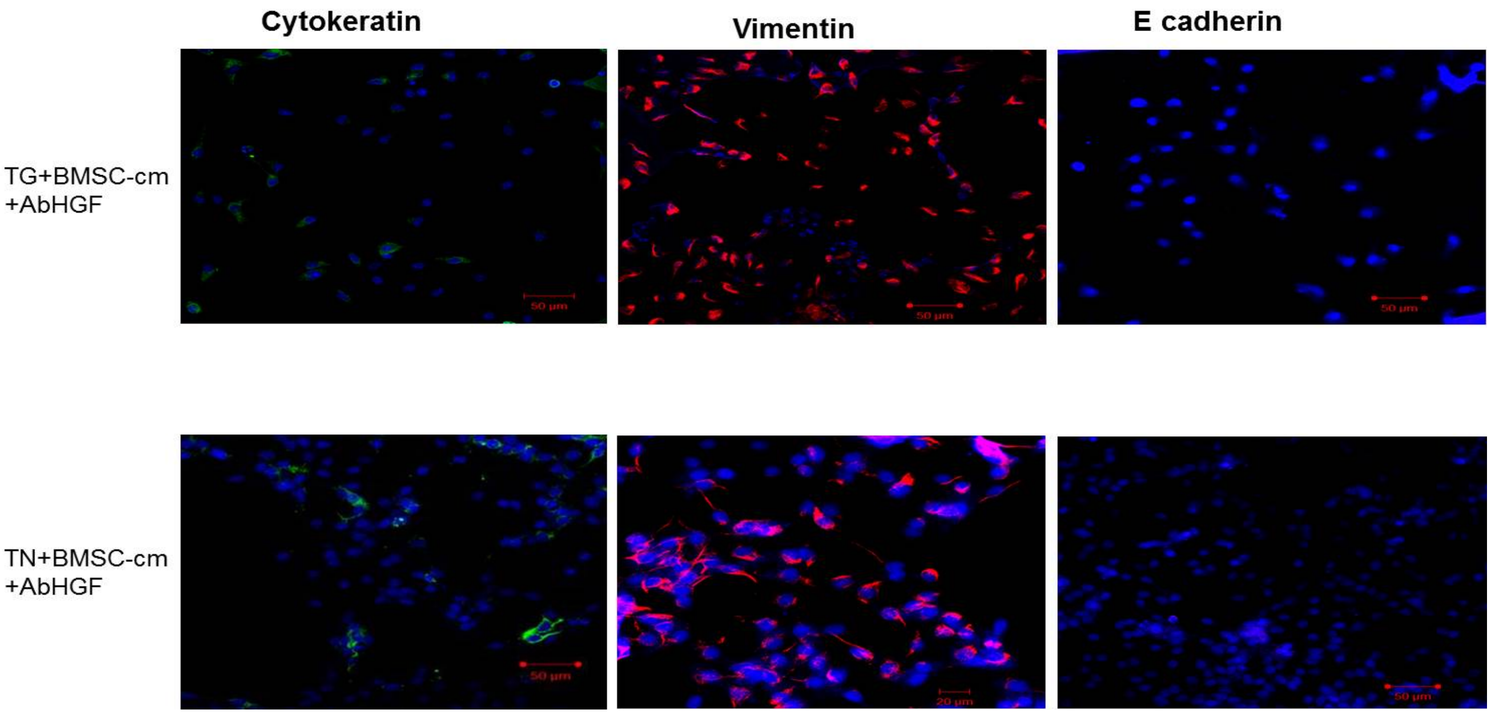

Supp Fig 5

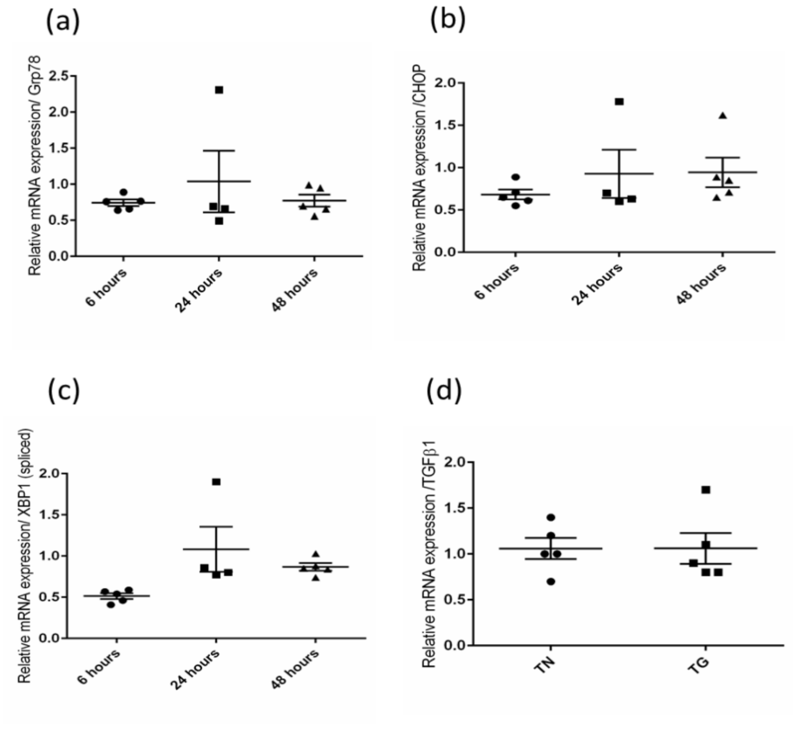

Supp Fig 6

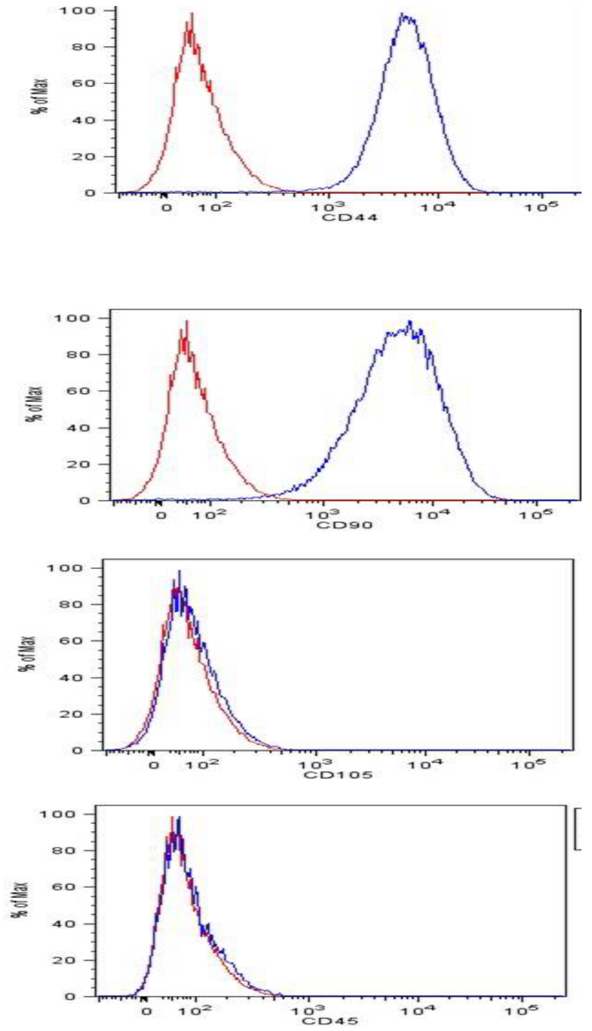

Supp Figure legend

Fig S 1) Primary alveolar epithelial cells from IPF patients have increased expression of surfactant protein c (SPc) after treatment with BMSC-cm, however when HGF is neutralized the SPc expression level is reduced again. Experiments were performed in triplicates (n=3).

Fig S 2) Western blot analysis performed to investigate the possible mechanism reveals increased autophosphorylation of the c-MET receptor and phosphorylation of its downstream signaling molecules upon BMSC-cm treatment, this effect being more prominent in the TG-induced A549 cells. Recombinant human HGF (rhHGF) and anti-HGF treatments were used as controls for c-MET signaling activation and inhibition, respectively. Rab 11 serves as a loading control, densitometry values are normalized to Rab11 expression). Densitometry values of pMET, pAKT and pERK were normalized to Rab11 and are presented graphically. pMET protein expression increased after BMSC-cm treatment in TG stressed A 549 cells, however no change was observed in TN stressed A549 cells. After neutralizing HGF the pMET levels were reduced too. pAKT expression was increased in both TN and TG stressed A549 cells after BMSC-cm treatment and were reduced after HGF neutralization. pERK expression was increased in TN stressed A 549 cells but no changes were observed in TG stressed cells after BMSC-cm treatment, however after HGF neutralization the pERK levels were reduced (Fig S2b). Total MET expression, in response to TN or TG was significantly reduced, and recovery was observed upon treatment with BMSC-cm. Total AKT did not show any significant change after ER stress however slight elevation was observed after BMSC-cm treatment. Total ERK 1 showed no alterations (Fig 2c). Additionally to understand the downstream pathways involved western blot analysis was performed for eIF2α and phosphorylated eIF2α. Both eIF2α and phosphorylated eIF2α were reduced after BMSC-cm treatment. In TN stressed cells the levels were reduced after rec HGF treatment too, however in TG treated cells no response was observed either with RecHGF or with Anti HGF antibody. Also caspase-3 expression and activity was reduced in TN stressed cells, however no activity was observed in TG treated cell (Fig 2c). b-actin serves as a control, densitometry values are normalized to b-actin expression and densitometry values are presented graphically (Fig2d). Experiments were performed in duplicate (n=2).

Fig S 3) Cell proliferation was reduced in ER stressed A549 cells and was significantly increased after treatment with BMSC-cm in both TN (S3a) and TG (S3b) stressed A 549 cells. Experiments were performed in triplicates (n=3).

Fig S 4) After HGF neutralization the expression of cytokeratin and E cadherin was reduced and vimentin was expressed again, as observed by immunofluroscence. (scale bar 20µm)

Fig S 5) A 549 cells were treated with TGFβ1, no increase in the mRNA expression of any ER stress markers was observed at 6 hours, 24 hours or 48 hours. Also, no change in mRNA expression of TGFβ1 levels were observed after TN or TG treatment of A 549 cells. Experiments were performed in triplicates (n=3).

Fig S 6) BMSC where characterized using known mesenchymal markers and cultured to collect conditioned media. The cells were negative for CD45 and stained positive for CD44, CD90 and for CD105.

Primers

Table S 1. Primer sequence of specific genes

GRp78	Forward CTG CCA TGG TTC TCA CTA AAA TG<br>Reverse TTA GGC CAG CAA TAG TTC CAG
XBP1 (spliced)	Forward TGG ATT CTG GCG GTA TTG AC<br>Reverse TCC TTC TGG GTA GAC CTCTG
CHOP	Forward GTA CCT ATG TTT CAC CTC CTG G<br>Reverse TGG AAT CTG GAG AGT GAG GG
PERK	Forward GAA CCA GAC GAT GAG ACA GAG<br>Reverse GGA TGA CAC CAA GGA ACC G
IRE1	Forward GCG AAC AGA ATA CAC CAT CAC<br>Reverse ACC AGC CCA TCA CCA TTG
ATF6	Forward CCT GTC CTA CAA AGT ACC ATG AG<br>Reverse CCT TTA ATC TCG CCT CTA ACC C
18S	Forward CGCCGCTAGAGGTGAAATTCT<br>Reverse CATTCTTGGCAAATGCTTTCG
hSPC	Forward CCTTCTTATCGTGGTGGTGGT<br>Reverse TCTCCGTGTGTTTCTGGCTCAT
TGFb1	Forward TCGAGTCGACCCTGCACAGCTCCAGGCACC<br>Reverse AATTGAATTCTGCTCCACCTTGGGCTTGCG
